# Systems-Based Approaches to Probing Metabolic Variation within the *Mycobacterium tuberculosis* Complex

**DOI:** 10.1371/journal.pone.0075913

**Published:** 2013-09-17

**Authors:** Emma K. Lofthouse, Paul R. Wheeler, Dany J. V. Beste, Bhagwati L. Khatri, Huihai Wu, Tom A. Mendum, Andrzej M. Kierzek, Johnjoe McFadden

**Affiliations:** 1 Animal Health and Veterinary Laboratories Agency (Weybridge), Department for Bovine Tuberculosis, New Haw, Surrey, United Kingdom; 2 Department of Microbial and Cellular Sciences, Faculty of Health and Medical Sciences, University of Surrey, Stag Hill, Guildford, Surrey, United Kingdom; University College Dublin, Ireland

## Abstract

The *Mycobacterium tuberculosis* complex includes bovine and human strains of the tuberculosis bacillus, including *Mycobacterium tuberculosis, Mycobacterium bovis* and the *Mycobacterium bovis* BCG vaccine strain. *M. bovis* has evolved from a *M. tuberculosis*-like ancestor and is the ancestor of the BCG vaccine. The pathogens demonstrate distinct differences in virulence, host range and metabolism, but the role of metabolic differences in pathogenicity is poorly understood. Systems biology approaches have been used to investigate the metabolism of *M. tuberculosis*, but not to probe differences between tuberculosis strains. In this study genome scale metabolic networks of *M. bovis* and *M. bovis* BCG were constructed and interrogated, along with a *M. tuberculosis* network, to predict substrate utilisation, gene essentiality and growth rates. The models correctly predicted 87-88% of high-throughput phenotype data, 75-76% of gene essentiality data and *in silico*-predicted growth rates matched measured rates. However, analysis of the metabolic networks identified discrepancies between *in silico* predictions and *in vitro* data, highlighting areas of incomplete metabolic knowledge. Additional experimental studies carried out to probe these inconsistencies revealed novel insights into the metabolism of these strains. For instance, that the reduction in metabolic capability observed in bovine tuberculosis strains, as compared to *M. tuberculosis*, is not reflected by current genetic or enzymatic knowledge. Hence, the *in silico* networks not only successfully simulate many aspects of the growth and physiology of these mycobacteria, but also provide an invaluable tool for future metabolic studies.

## Introduction

The pathogenic microorganisms constituting the *Mycobacterium tuberculosis complex* are associated with important human and animal diseases. *M. bovis* is the causative agent of bovine tuberculosis, a chronic and occasionally fatal infectious disease primarily infecting cattle and other livestock; but is capable of infecting a wide range of mammals and other vertebrates, including humans [1,2]. Bovine tuberculosis causes immense economic loss in many countries, either from loss of livestock, disease testing, or compensation. Worldwide, agricultural losses are estimated to be around $3 billion a year [3]. *M. bovis* is very closely related to *M. tuberculosis*, a virulent tubercle bacillus estimated to infect a third of the world’s population and cause the deaths of 1.4 million people each year [4]. In an attempt to prevent tuberculosis infections more than 3 billion individuals [5] have been immunised with *M. bovis* BCG, a live attenuated derivative of *M. bovis.*

*M. tuberculosis*, *M. bovis* and *M. bovis* BCG are characterised by 99.9% similarity at the nucleotide level [3,5,6]. However, the genetic deletions, rearrangements and duplications that *M. bovis* and *M. bovis* BCG have undergone relative to *M. tuberculosis* results in widely differing host tropisms, phenotypes and pathogenicity [2,3,5-9]. Whilst large deletions, such as the regions of difference identified in *M. bovis* and *M. bovis* BCG, have been shown to encode virulence factors and result in attenuation of infection [9-12], the genetic basis of these profound variations are mostly undefined. Defining the metabolic differences between the three species is of particular importance as metabolic adaptation to the host environment has been highlighted as a key component of the pathogenic strategy of *M. tuberculosis* [13-17] and is also likely to be important for the virulence of *M. bovis*.

Previous targeted studies have identified the genetic basis of some observed metabolic differences, for example, the inability of *M. bovis* to generate energy from glycolytic intermediates [3,18,19]. This defect is thought to be due to the inactivation of pyruvate kinase which causes a disconnection in central metabolism between glycolysis and the Tricarboxylic acid cycle (TCA cycle) [18,19]. However, although there have been focused investigations into the metabolic differences between the human and bovine tubercle bacillus, systems level comparisons of metabolism have not yet been undertaken.

A systems biology approach provides very effective methods for studying metabolism. Genome scale metabolic reaction networks incorporate all known biochemical reactions within a cell and represent a global system where reaction pathways are defined within the context of whole-cell metabolism. These networks are able to predict phenotypic behaviour, aid in hypothesis generation, identify missing reactions and provide information on the robustness of the metabolic networks, which can be used to identify vulnerable pathways that may be targeted with novel drugs. The creation of *M. tuberculosis* genome scale reaction networks [20-23] has provided a mechanism to study its metabolism in a systemic manner and a basis for the modelling and metabolic comparison of *M. bovis* and *M. bovis* BCG strains.

In this study we present the first genome scale metabolic networks for *M. bovis* and *M. bovis* BCG, along with a phenotypic analysis of *M. tuberculosis*, *M. bovis* and *M. bovis* BCG. The networks are freely available in the Supporting Information (Tables S1-S3; Models S1-S3) and online for use with our interactive software (http://sysbio.sbs.surrey.ac.uk/) [24]. To our knowledge this is the first time the metabolism of a pathogen and its vaccine strain have been compared on a systems level and any identified differences have the potential to aid investigations into causes of *M. bovis* BCG attenuation and the suitability of novel vaccines. The models qualitatively predict phenotype data with between 87-88% agreement, gene knockout results with 75-76% accuracy, and quantitative assessment of measured carbon uptake rates as a function of growth rate show that *in silico* growth rates are comparable to *in vitro* results. Therefore, these reaction networks successfully simulate many aspects of mycobacterial metabolism and can be used to examine the metabolic differences between these strains.

## Materials and Methods

### Bacterial strains and growth conditions

*M. tuberculosis* H37Rv, *M. bovis* AF2122/97 and *M. bovis* BCG Pasteur were used for this study. Frozen stocks were maintained in 10% (vol/vol) glycerol at −80°C. Middlebrook 7H9 broth containing 5% (vol/vol) albumin-dextrose-catalase enrichment medium supplement (ADC) (Becton Dickenson), 2 g/L pyruvate and 0.05% (vol/vol) Tween 80 was used to grow cultures from frozen stocks at 37°C, either statically or rolling. Brain heart infusion agar was used to assess culture purity (Becton Dickinson).

For the Roisin’s agar experiments cultures were grown until late exponential phase (OD_600_ = 1.0) in 7H9 containing ADC, pyruvate and Tween 80 (as described), washed twice with Ringer’s solution with 0.2% (vol/vol) tyloxopol and plated in triplicate onto Roisin’s agar containing sole carbon and nitrogen sources. When testing sole carbon source assimilation, cells were grown on Roisin’s minimal media [25] with 10 g/L agarose containing sole carbon sources at 5 g/L (2-oxoglutarate, L-alanine, D-arabinose, L-arginine, L-asparagine, L-aspartic acid, citrate, D-fructose, fumarate, D-galactose, D-glucose, L-glutamate, L-glutamine, glycerol, glycine, L-histidine, L-isoleucine, D-lactose, L-leucine, L-lysine, malate, D-maltose, D-mannose, L-methionine, L-phenylalanine, L-proline, propanoate, D-raffinose, D-rhamnose, D-ribose, L-serine, D-serine, succinate, D-sucrose, L-threonine, D-trehalose, L-valine, D-xylose). When testing sole nitrogen source assimilation Roisin’s minimal media was used with 5 g/L pyruvate, 10 g/L agarose and 5.9 g/L sole nitrogen source (L-alanine, ammonia, L-arginine, L-asparagine, L-aspartic acid, L-cysteine, L-glutamate, L-glutamine, glycine, L-histidine, L-isoleucine, L-leucine, L-lysine, L-methionine, L-phenylalanine, L-proline, L-serine, D-serine, L-threonine, L-tryptophan, L-tyrosine, urea, L-valine). Plates were incubated for up to 12 weeks. To test for any mutations which may have given false results any positives after 6 weeks were independently tested a further two times.

For growth curves inoculum cultures of *M. bovis* were grown statically until late exponential phase (OD_600_ = 1.0) in Roisin’s minimal media with 5 g/L pyruvate and 0.2% (vol/vol) tyloxapol. Using a 1% inoculum 100 ml rolling (2 rpm) cultures were set up in Roisin’s minimal media with 5 g/L D-glucose, 0.2% (vol/vol) Tween 80 or 5 g/L D-glucose and 0.2% (vol/vol) Tween 80 as carbon sources. A Biomate, ThermoSpectronic spectrophotometer was used to take daily OD readings until they started to fall, presumably due to bacterial death.

### Construction of GSMN-MB and BCG

The first *M. tuberculosis* genome scale metabolic network [20] (GSMN-TB) (Table S1; Model S1) created for the H37Rv strain formed the basis for the reconstruction of *M. bovis* AF2122/97 (GSMN-MB) (Table S2; Model S2) and *M. bovis* BCG Pasteur (GSMN-BCG) (Table S3; Model S3). Genolist [26] was used to assign respective *M. bovis* and *M. bovis* BCG gene numbers to the genes present in GSMN-TB and alter annotations which since the GSMN-TB was published have been assigned with an alternative enzymatic function. Using data compiled by Genolist [26] any mutations between *M. tuberculosis*, *M. bovis* and *M. bovis* BCG leading to changes in protein sequence were identified. Published scientific literature was used to investigate these genes and to identify metabolic differences not caused by DNA mutations within enzyme sequences. This information was used to alter the networks to reflect *M. bovis* or *M. bovis* BCG metabolism (Table S4).

### Biolog phenotype data

Biolog Phenotype MicroArray [27] experiments which examined the ability of *M. tuberculosis* H37Rv and *M. bovis* AF2122/97 to respire in the presence of 190 carbon and 95 nitrogen sources (PM1, 2a, 3b [27]), were obtained from Khatri, B et al. 2013 [8]. Equivalent experiments for *M. bovis* BCG were carried out using the described method [8]. Three to four biological replicates were performed for each plate and a cell free control plate was also included to test for abiotic dye reduction. The raw data was analysed using OmniLog PM kinetic plot software [27], with final colour intensity values and manual analysis of kinetic curves used to assess respiration. Wells were considered positive if the final colour intensity value minus the negative control and minus the standard deviation of the negative controls was a positive value [28]. Results were verified by analysing the kinetic curves. Substrates considered to be respired had at least 50% of duplicate wells showing a positive result. The confidence levels applied to the data were determined by the number of concordant results for each biological replicate [28]. High, medium and low confidence results were categorised as substrates where 100%, >75% and >50% of replicates produced the same result.

### Modelling of phenotype experiments

The composition of Biolog base medium [27] is not published, however, the mycobacteria were not able to respire in this medium without the added carbon or nitrogen source. To model the carbon source experiment we therefore simulated the media as a modified form of Roisin’s minimal media [25] containing unlimited quantities of ammonia, phosphate, iron, sulfate, carbon dioxide and a Biolog carbon source influx of 1 mmol/g DWt/h. Similarly the nitrogen source experiment was simulated using a modified form of Roisin’s media, where ammonia was replaced with 1 mmol/g DWt/h of the Biolog nitrogen source and pyruvate was used as a carbon source (influx at 1 mmol/g DWt/h). Flux balance analysis (FBA) was performed under aerobic conditions using biomass as the objective function.

### Radioactive glucose uptake in *M. bovis* AF2122/97 and *M. tuberculosis* H37Rv

Cultures of *M. tuberculosis* and *M. bovis* were grown until exponential phase (OD_600_ 0.6-0.8) in Roisin’s minimal media with 5 g/L pyruvate and 0.2% (vol/vol) tyloxapol, washed twice and then resuspended in Roisin’s media (no carbon, tyloxapol). 1 ml of culture was added to a universal tube containing 3700 becquerels of [6-^14^C] D-glucose and 1 ml of 1 M sodium hydroxide and incubated for 4 h at 37°C. Samples were carried out in triplicate with negative controls (identically prepared heat killed suspensions) tested in parallel. Samples were filtered through Whatman GFC glass microfibre filters, washed 3 times with 0.025% (vol/vol) tyloxapol and placed in scintillation tubes with 250 µl of 70% ethanol and 5 ml of BDH Scintran Fluoran flow scintillation fluid. The sodium hydroxide was directly added to the scintillation fluid. Radioactivity was counted using a Packman tri-carb liquid scintillation counter and dpm calculated from a quench curve. Dpm for negative controls were subtracted from dpm for the live samples before the mean average uptake was calculated. For the analysis of [6-^14^C] D-glucose radioactivity incorporation in *M. bovis* when both [6-^14^C] D-glucose and non-radiolabelled Tween 80 were available as carbon sources, the experiment carried out as described above, except that the incubation medium was supplemented with 0.2% (vol/vol) Tween 80.

### Modelling of growth rate experiments

To model *in vitro* growth rate experiments in GSMN-BCG and TB the media was simulated as a modified form of Roisin’s minimal media (see modelling of phenotype experiments) with carbon sources constrained to experimentally derived values [29] and the biomass composition for slow or fast growth used as the objective function.

### Gene essentiality predictions and comparison with TRASH and deep sequencing data

The maximal theoretical growth rate of each *in silico* gene knock out was calculated by removing single genes from the network and performing FBA linear programming as described in Beste et al., 2007 [20]. The computational predictions were compared to the gene essentiality findings of Transposon site hybridization (TraSH) mutagenesis [30] and deep sequencing [31] experiments.

## Results

### The genome-scale metabolic networks of *M. bovis* and *M. bovis* BCG

The genome scale metabolic network of *M. tuberculosis*, GSMN-TB [20], (Table S1; Model S1) was used as a starting point for the reconstruction of the genome scale networks of *M. bovis* (Table S2; Model S2) and *M. bovis* BCG (Table S3; Model S3). Reactions were adapted to reflect *M. bovis* and *M. bovis* BCG reactions based on genome annotations, protein sequences and published biochemical data [3,5,6,18,26,32-49] (Table S4). For orthologous genes GSMN-TB gene assignments were changed to *M. bovis* and *M. bovis* BCG gene numbers, with each DNA sequence analysed for changes relative to *M. tuberculosis*. The analysis identified 228 and 204 genes with non-synonymous sequence differences in *M. bovis* and *M. bovis* BCG and these genes catalysed approximately 30% of reactions within each network. Only a small proportion (~19%) of sequence differences were predicted to lead to metabolic differences requiring network modifications, the vast majority of which were the removal of genes and reactions (Table 1). For GSMN-MB 42 genes and 14 reactions were deleted from the original GSMN-TB network, whilst slightly fewer genes (40) and more reactions (16) were deleted from the GSMN-BCG. These alterations appear to support the theory that *M. bovis* has evolved from a progenitor of the *M. tuberculosis* complex and that *M. tuberculosis* is more closely related to this common ancestor [50].

**Table 1:** Changes to the GSMN-TB network to create GSMN-MB and GSMN-BCG.

Gene	GSMN-TB	GSMN-MB	GSMN-BCG
Glycerol kinase (*glpK*)	1	-	1
Glycerol-3-phosphate dehydrogenase (*glpD*)	2	2	3
GDP-D-rhamnose biosynthesis (*gca*, *gmdA*)	2	1	1
GDP-4-dehydro-6-deoxy-D-mannose epimerase	1	0	0
UTP-hexose-1-phosphate uridylyltransferase (*galT*)	2	1	1
β-glucosidase (*bglS*)	1	-	-
Pyruvate kinase (*pykA*)	1	-	1
Isocitrate lyase (icl)	1	2	2
(S)-2-hydroxy-acid oxidase	1	-	-
Nitrate reductase (*nar*)	5	-	-
Fumarate reductase (frd)	8	7	7
Glycine dehydrogenase (*gcvB*)	<=>	=>	=>
L-serine ammonia-lyase (*sdaA*)	1	1	-
Alanine dehydrogenase (*ald*)	1	-	-
Nicotinamidase	1	-	-
Precorrin-6Y C5,15-methyltransferase	1	-	-
Molybdopterin biosynthesis protein (*moaE*)	3	-	-
enoyl-CoA hydratase/isomerases (*echA*)	21	21	21
Phospholipases	28	23	23
Methoxy mycolic acid synthase (*mmaA3*)	1	1	-
Synthesis of methoxy mycolic acids	Biomass	Biomass	
Polyketide synthase (*pks15/1*)	-	1	1
Glycosyltransferases	2	-	-
Sulfotransferases	3	2	3
Sulfolipid-1 synthesis	1	-	-
Mas-like gene (*msl3*, *msl4*, *msl5*)	4	3	3
Nitrate transporter (*narK2*)	1	-	-
Phosphate transport via ABC system	8	-	-
Glycerol-3-phosphate antiporter (*ugp*)	4	-	-
Sulfolipid-1	Biomass		
Mycoside b		Biomass	Biomass
Triacylglycerol synthases	15	14	14

Numerical value Number of genes catalysing the reaction; - indicates reaction is deleted from the network; 0 indicates an orphan reaction

=> Irreversible reaction

<=> Reversible reaction

Biomass Required for Biomass production

The constructed metabolic networks for both *M. bovis* and *M. bovis* BCG (Table 2) therefore contain fewer reactions, metabolites and genes than an updated GSMN-TB network, GSMN-TB 1.1. GSMN-TB 1.1 includes some corrections to the original GSMN-TB, plus additional pathways, such as cholesterol metabolism, that were not implemented in the earlier published version [20]. Due to the reduction in genes within the networks the *M. bovis* and *M. bovis* BCG models have a slightly higher fraction of essential genes (31% and 30% respectively; Tables S5 and S6) compared to GSMN-TB 1.1 (29%; Table S7). These values are slightly lower than the predicted value of 35% essential genes in the entire *M. tuberculosis* genome but within the 95% confidence interval (28-41%) [51]. All three GSMN networks are available in the Supporting Information (Tables S1-S3; Models S1-3) and online for use with our interactive software (http://sysbio.sbs.surrey.ac.uk/) [24].

**Table 2:** Statistics of the mycobacterial reaction networks.

	Reaction network		
Reaction Class	GSMN-TB 1.1	GSMN-MB	GSMN-BCG
Total number of reactions	876	863	861
Cytosolic reactions	745	735	733
Transport reactions	131	128	128
Genes	759	718	720
Orphan reactions	198	200	200
Total number of metabolites	766	757	754
Internal metabolites	667	660	657
External metabolites	99	97	97

Although the GSMN-MB and BCG models gave very similar predictions to the GSMN-TB 1.1, some interesting differences between *in silico* predictions and published experimental data [3,18,19,33,44] were found. GSMN-MB was able to utilise carbohydrates such as glucose *in silico*, although *in vitro M. bovis* is actually unable to grow on these substrates [18,19] (discussed further below). Another area where *in silico* predictions did not accord with experimental data was in amino acid synthesis. In *M. tuberculosis*, alanine dehydrogenase catalyses the oxidative deamination of L-alanine or, in the reverse direction, the reductive amination of pyruvate to yield alanine, but this activity is lost [33,44] in *M. bovis* and *M. bovis* BCG due to a frameshift caused by single base pair deletion [3]. Deletion of alanine dehydrogenase from the bovine networks resulted in a non-feasible network (no growth) unless L-alanine was supplied as a substrate, since this is the only biosynthetic route leading to L-alanine in the network. Yet, in contrast to the predictions *M. bovis* and *M. bovis* BCG are not alanine auxotrophs. Alanine dehydrogenase knockout mutants of *M. tuberculosis* are also not alanine autotrophs [19] and ^13^C labelling experiments detected identical alanine labelling patterns [29] for *M. tuberculosis* and *M. bovis* BCG; indicating that an alternative pathway for alanine synthesis must be active in all three strains. To preserve alanine prototrophy, the alanine dehydrogenase reaction has therefore been retained in the GSMN-MB and BCG models as an irreversible orphan reaction [19,33,44].

### Validation of the model by comparison with Biolog phenotype data

The Biolog [27] high throughput phenotyping system was utilised to obtain additional insight into metabolic capability of *M. bovis* and *M. bovis* BCG and to further test the predictive accuracy of the GSMN-MB and GSMN-BCG networks. For comparison, we also examined *M. tuberculosis*. Biolog [27] is a commercially available phenotype microarraying platform that is capable of high-throughput screening for the ability to utilise a large number of substrates. It employs the reduction of tetrazolium dye by NADH as a reporter system for measuring respiration [27]. Respiration is of course different from growth, as predicted by the network models; but our working assumption was that some degree of growth has to occur to reduce the respiratory substrate sufficiently to see a positive reaction.

Carbon substrate utilisation for *M. tuberculosis*, *M. bovis* and *M. bovis* BCG is presented in Table 3 (showing only substrates metabolised by at least one strain). Of the 190 carbon sources tested (Tables S8-S10) 33 substrates were metabolised by at least one of the mycobacteria tested, with 17 utilised by all three. These substrates included amino acids, TCA cycle intermediates, sorbitan derivatives, 3 carbon compounds and hexose or hexose containing carbohydrates. *M. tuberculosis* was able to respire more of these substrates (27) than *M. bovis* (25) or *M. bovis* BCG (22). Of the 95 nitrogen sources tested (Tables S11-S13) only 13 were able to be utilised as sole nitrogen sources (Table 4) by at least one of the three strains and these were mainly amino acids. Unlike carbon source data, the number of nitrogen sources utilised was similar between the three species.

**Table 3:** Carbon substrates utilised by *M. tuberculosis*, *M. bovis* and *M. bovis* BCG.

	*M. tuberculosis*			*M. bovis*			*M. bovis* BCG		
Substrates	Biolog	Roisin’s agar	*in silico*	Biolog	Roisin’s agar	*in silico*	Biolog	Roisin’s agar	*in silico*
2-oxoglutarate	C	C	C	-	C>	C	-	C	C
Acetate	C	C	C	C	C	C	C	C	C
Acetoacetic acid	C	NT	-	C	NT	-	C	NT	-
Adenosine	-	NT	C	-	NT	C	C	NT	C
D-alanine	C	NT	C	-	NT	-	-	NT	-
L-alanine	C	C	C	-	-	-	-	-	-
L-asparagine	C	C	C	-	C	C	C	C	C
Butyric acid	C	NT	-	C	NT	-	C	NT	-
Caproic acid	C	NT	C	C	NT	C	C	NT	C
Citrate	C	C	C	C	C	C	C	C	C
D-fructose-6-phosphate	C	NT	C	C	NT	C	-	NT	C
D-glucose-6-phosphate	C	NT	C	-	NT	C	C	NT	C
D-glucose	C	C	C	C	-	C	-	C	C
L-glutamate	C	C	C	C	C	C	C	C	C
L-glutamine	C	C	C	-	C	C	-	C	C
Glycerol	C	C	C	C	-	C	C	C	C
Glycine	C	C	C	-	C	C	-	C	C
L-lactate	C	NT	C	C	NT	C	C	NT	C
D-malic acid	-	NT	C	C	NT	C	C	NT	C
L-malic acid	C	C	C	C	C	C	C	C	C
D-mannose	-	C	C	C	C	C	-	C	C
Methyl-pyruvate	C	NT	-	C	NT	-	C	NT	-
Mono methyl-succinate	C	NT	-	C	NT	-	C	NT	-
N-acetyl-glucosamine	-	NT	-	C	NT	-	-	NT	-
Oxalomalic acid	C	NT	-	C	NT	-	C	NT	-
Propanoate	-	C	C	C	C	C	C	C	C
Pyruvate	C	C	C	C	C	C	C	C	C
D-serine	C	-	-	C	-	-	-	-	-
D-tagatose	-	NT	-	C	NT	-	-	NT	-
D-trehalose	C	C	C	C	C	C	C	C	C
Tween 20	C	NT	C	C	NT	C	C	NT	C
Tween 40	C	NT	C	C	NT	C	C	NT	C
Tween 80	C	C	C	C	C	C	C	C	C

C Utilised as a carbon substrate - Not utilised as a carbon substrate NT Not tested

**Table 4:** Nitrogen substrates utilised by *M. tuberculosis*, *M. bovis* and *M. bovis* BCG.

	*M. tuberculosis*			*M. bovis*			*M. bovis* BCG		
Substrate	Biolog	Roisin’s agar	*in silico*	Biolog	Roisin’s agar	*in silico*	Biolog	Roisin’s agar	*in silico*
L-Alanine	N	N	N	-	-	-	-	-	-
Allantoin	-	NT	-	N	NT	-	-	NT	-
L-Asparagine	N	N	N	N	N	N	N	N	N
L-Aspartic Acid	-	N	N	-	N	N	N	N	N
L-Cysteine	N	-	-	N	N	-	N	-	-
D-Galactosamine	N	NT	-	N	NT	-	N	NT	-
D-Glucosamine	-	NT	-	N	NT	-	-	NT	-
L-Glutamic Acid	N	N	N	N	N	N	N	N	N
L-Glutamine	N	N	N	N	N	N	N	N	N
L-Ornithine	N	NT	-	N	NT	-	N	NT	-
D-Serine	N	-	-	N	-	-	N	-	-
L-Serine	N	N	N	N	N	N	-	-	N
L-Threonine	-	N	N	-	-	N	N	-	N

N Utilised as a nitrogen substrate - Not utilised as a nitrogen substrate NT Not tested

Analysis of *in silico* predictions verses Biolog data showed that all three networks qualitatively predicted the Biolog data with a similar overall accuracy. For GSMN-TB 1.1, GSMN-MB and GSMN-BCG simulations 84%, 81% and 84% of substrates matched Biolog results respectively (Tables S8-S13). Substrate analysis identified results that appeared anomalous with previous studies [18,19,33,44,52]. For instance, *M. bovis* tested positive for glucose and glycerol respiration, however, as mentioned above, *M. bovis* is unable to utilise carbohydrates including glucose and glycerol as sole carbon sources [18,19]. Similarly, many *in silico* predictions for amino acid utilisation did not correlate with experimental data [33,44,52]. To independently probe the discordant results *M. tuberculosis*, *M. bovis* and *M. bovis* BCG were grown on minimal Roisin’s agar media containing sole carbon or nitrogen sources.

Overall (Tables S8-S13) *in vitro* experiments using Roisin’s agar media resolved many of the inconsistencies between *in silico* predictions and the Biolog data. For instance, in accordance with expectations, *M. bovis* was unable to grow on either glucose or glycerol when provided as the sole carbon source in Roisin’s agar. The anomalous positive Biolog results may be due to the small amount of Tween 80 present in the Biolog base media. It has been shown previously [8,53], and confirmed here (see below), that Tween 80 and glucose are used synergistically for growth by *M. bovis* strains.

When the anomalous Biolog results were corrected using Roisin’s agar data the GSMN-MB model accurately predicts 87% of phenotypes studied, whilst the GSMN-BCG predicts 88% correctly. The GSMN-TB 1.1 is slightly more accurate (91%) at simulating cellular phenotypes than the bovine networks due to false positive *in silico* growth predictions for *M. bovis* and *M. bovis* BCG. In this analysis results generated on Roisin’s agar media were used instead of Biolog results when the outcomes differed because growth on agar plates assesses biomass production rather than respiration. Therefore, growth on Roisin’s agar media more accurately tests *in silico* predictions of growth than Biolog experiments.

### Analysis of glucose metabolism

**Figure 1:**
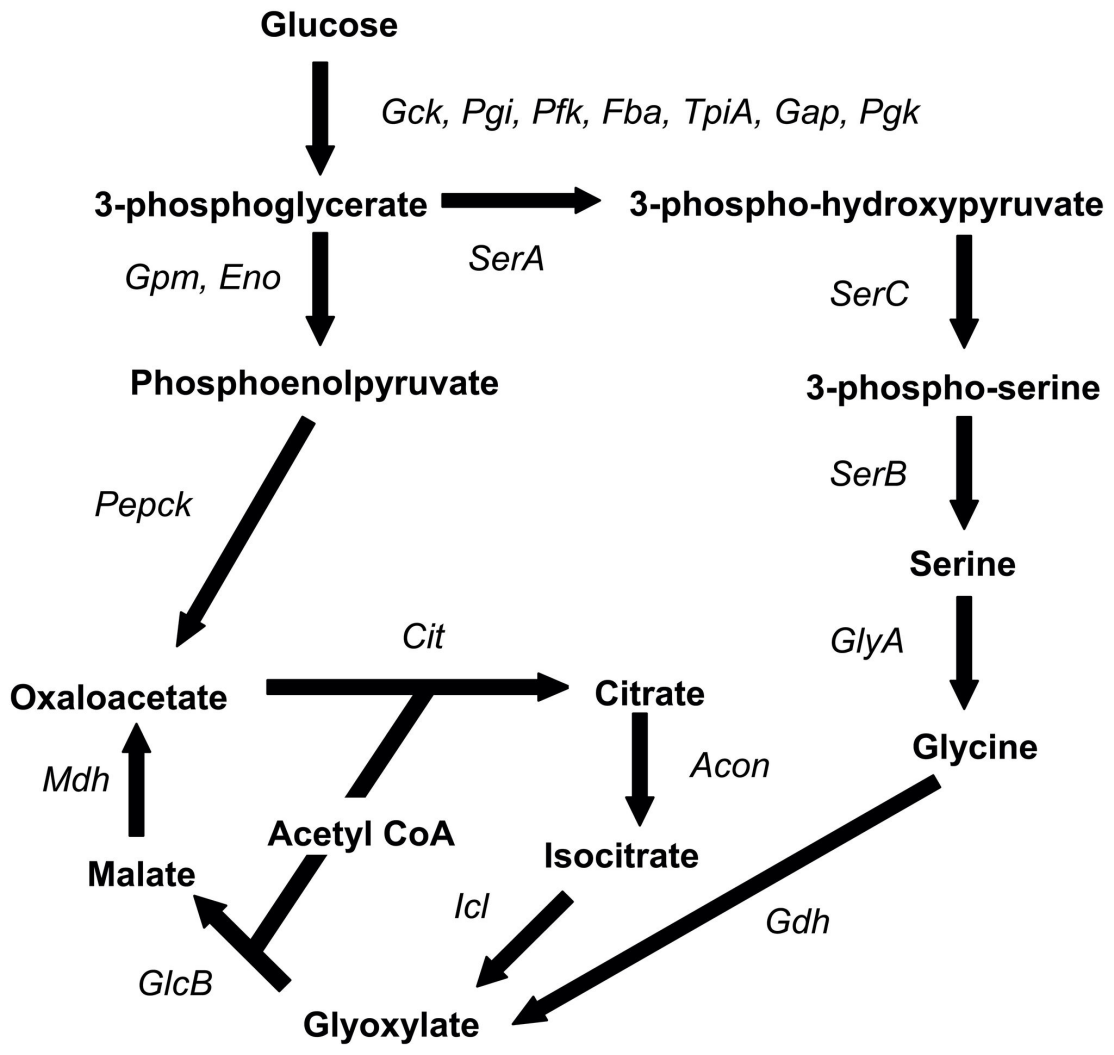
GSMN-MB *in silico* flux prediction when glucose is a sole carbon source. The *in silico* prediction of flux from glucose to the TCA cycle when glucose is a sole carbon source for *M. bovis*. *Acon*: aconitase, *Cit*: citrate synthase, *Eno*: enolase, *Fba*: fructose-bisphosphate aldolase, *Gck*: glucokinase, *Gdh*: glycine dehydrogenase, *GlcB*: malate synthase, *GlyA*: glycine hydromethytransferase, *Gpm*: phosphoglycerate mutase, *Icl*: isocitrate lyase, *Mdh*: malate dehydrogenase, *Pepck*:, phosphoenolpyruvate carboxykinase, *Pfk*: 6-phosphofructokinase, *Pgi*: glucose-6-phosphate isomerase, *SerA*: phosphoglycerate dehydrogenase, *SerB*: phosphoserine phosphatase, *SerC*: phosphoserine transaminase, *TpiA*: triose-phosphate isomerase.

GSMN-MB incorrectly predicts that *M. bovis* should grow on glucose as a sole carbon source. *In silico*, glucose enters the central metabolism of *M. bovis* via the glycolytic pathway and bypasses the blocked pyruvate kinase connection between glycolysis and the TCA cycle via either the anaplerotic/gluconeogenic enzyme, phosphoenolpyruvate carboxykinase, that interconverts phosphoenolpyruvate (glycolytic intermediate) and oxaloacetate (TCA cycle intermediate) or a serine-glycine pathway (phosphoglycerate dehydrogenase, phosphoserine transaminase, phosphoserine phosphatase, glycine hydroxymethyltransferase and glycine dehydrogenase (Figure 1)). The hypothesis is that a second metabolic or regulatory defect in glucose metabolism contributes to the *M. bovis* glucose phenotype, in addition to the inactive pyruvate kinase [18,19].

To further explore *M. bovis* glucose metabolism, *M. bovis* and *M. tuberculosis* were incubated with [6-^14^C] D-glucose as the only available carbon source for four hours. *M. bovis* could uptake a small amount of the labeled glucose but the rate was 16-fold less than *M. tuberculosis*, consistent with a defect in glucose uptake (Table 5). Interestingly, supplementation of the glucose media with Tween 80 significantly stimulated glucose uptake by *M. bovis* (2.5 times). However, the fate of this additional glucose was not oxidation to carbon dioxide as the amount of ^14^C-CO_2_ generated was unchanged by the Tween 80 supplementation. Maximum growth rates and OD_600_ achieved on these substrates also indicated synergistic interaction [8] as growth of *M. bovis* on Roisin’s media with glucose and Tween 80 exceeded that achieved with either compound alone (Table 5).

**Table 5:** Glucose uptake experiments in *M. bovis* and *M. tuberculosis*

	Becquerels per mg dry weight			Growth rates on non-radiolabelled substrates		
	*M. tuberculosis*	*M. bovis*				
Carbon substrates	Assimilated	Assimilated	CO_2_ evolved	Maximum growth rate		Maximum OD
[6-^14^C] D-glucose	56.6 +/- 3.2	3.5 +/- 0.9	1.0 +/- 0.2	-		0.015 +/- 0.004
Tween 80	N/A	N/A	N/A	0.007 +/- 0.001		0.545 +/- 0.044
[6-^14^C] D-glucose and Tween 80	N/A	8.7 +/- 0.7	1.0 +/- 0.3	0.013 +/- 0.001		1.171 +/- 0.066

### Analysis of amino acid metabolism

Biolog data and *in silico* predictions were notably different for the utilisation of amino acids as sole carbon or nitrogen sources (Table 6) as the models predicted a greater metabolic potential than was actually demonstrated experimentally. Many of these discrepancies were resolved by the Roisin’s agar experiments, as more amino acids were shown to support growth in Roisin’s agar than were positive for respiration in Biolog. Possible reasons for this include large differences in the incubation times for these experiments. In support of this theory, the amino acids not utilised in the Biolog experiments predominantly produced dysgonic colonies with a lag time of around 6-8 weeks on Roisin’s agar (*M. bovis*: arginine C, N; glutamine C; glutamate C; glycine C; isoleucine C, N; proline C; serine C, N; *M. bovis* BCG: arginine C, N; glutamine C, N; glycine C; isoleucine C; proline C; serine C). Interestingly, the experimental data showed that the number of viable substrates decreased from *M. tuberculosis* to *M. bovis* to *M. bovis* BCG. This was not however reflected by significant network differences.

**Table 6:** The utilisation of amino acids as carbon and nitrogen sources by *M. tuberculosis*, *M. bovis* and *M. bovis* BCG

	*M. tuberculosis*						*M. bovis*						*M. bovis* BCG					
Substrate	Biolog		Roisin’s agar		in silico		Biolog		Roisin’s agar		in silico		Biolog		Roisin’s agar		in silico	
Alanine	C	N	C	N	C	N	-	-	-		-	-	-	-	-		-	-
Arginine	-	-	C	N	-	N	-	-	C	N	-	N	-	-	C	N	-	N
Asparagine	C	N	C	N	C	N	-	N	C	N	C	N	C	N	C	N	C	N
Aspartate	-	-	C	N	C	N	-	-	C	N	C	N	-	N	C	N	C	N
Cysteine	NT	N	-	-	-	-	NT	N	-	N	-	-	NT	N	-	-	-	-
Glutamate	C	N	C	N	C	N	C	N	C	N	C	N	C	N	C	N	C	N
Glutamine	C	N	C	N	C	N	-	N	C	N	C	N	-	N	C	N	C	N
Glycine	C	-	C	N	C	N	-	-	C	-	C	N	-	-	C	-	C	N
Histidine	-	-	-	-	-	-	-	-	-	-	-	-	-	-	-	-	-	-
Isoleucine	-	-	C	N	C	N	-	-	C	N	C	N	-	-	C	-	C	N
Leucine	-	-	-	-	-	-	-	-	-	-	-	-	-	-	-	-	-	-
Lysine	-	-	-	-	-	-	-	-	-	-	-	-	-	-	-	-	-	-
Methionine	-	-	-	-	-	-	-	-	-	-	-	-	-	-	-	-	-	-
Phenylalanine	-	-	-	-	-	-	-	-	-	-	-	-	-	-	-	-	-	-
Proline	-	-	C	N	C	N	-	-	C	-	C	N	-	-	C	-	C	N
Serine	-	N	C	N	C	N	-	N	C	N	C	N	-	-	C	-	C	N
Threonine	-	-	-	N	C	N	-	-	-	-	C	N	-	N	-	-	C	N
Tryptophan	NT	-	-	-	-	-	NT	-	-	-	-	-	NT	-	-	-	-	-
Tyrosine	NT	-	-	-	-	-	NT	-	-	-	-	-	NT	-	-	-	-	-
Valine	-	-	-	N	C	N	-	-	-	-	C	N	-	-	-	-	C	N

C Utilised as a carbon substrate N Utilised as a nitrogen substrate - No respiration/growth NT Not tested

**Figure 2:**
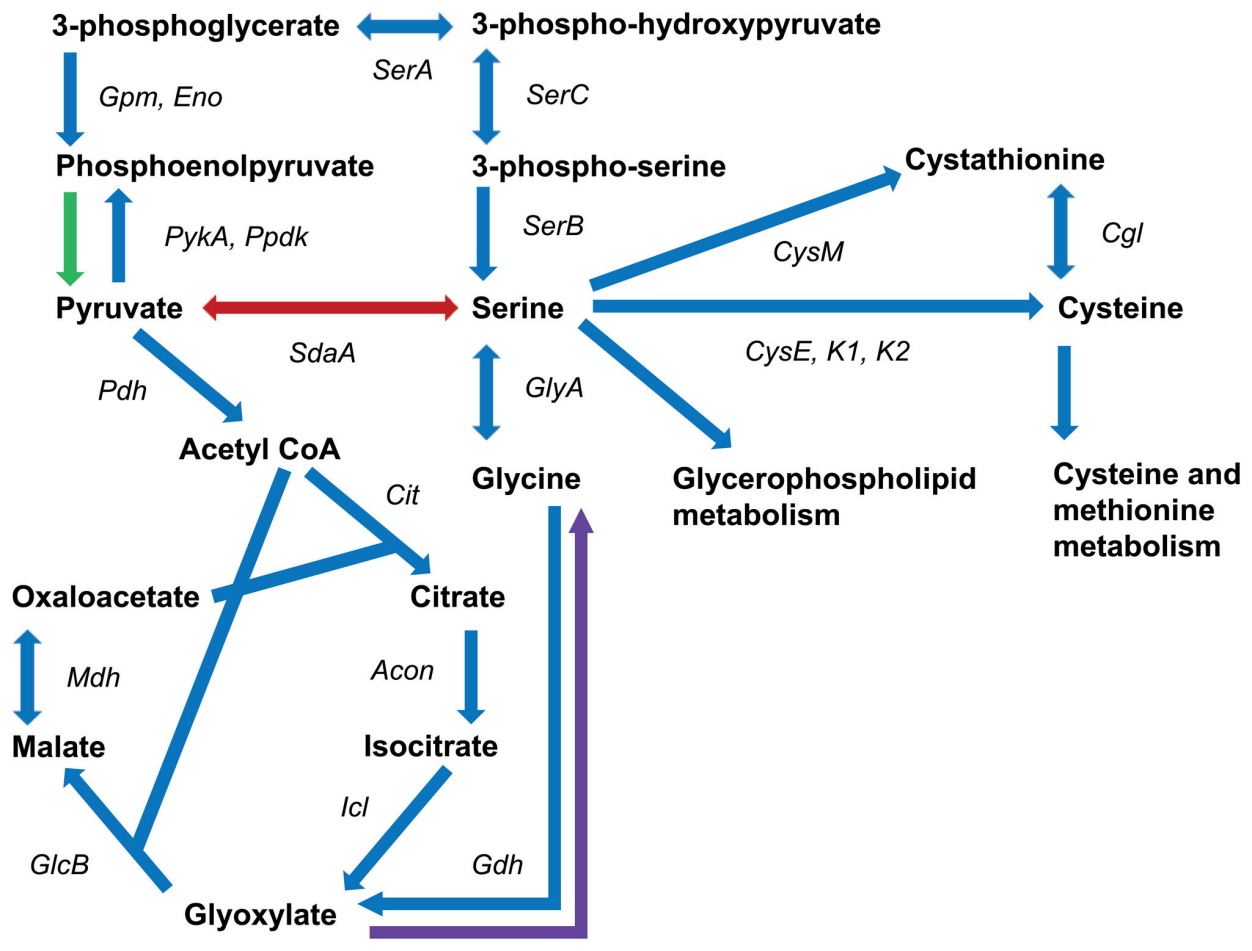
The metabolic pathways that converge around L-serine in Mycobacterium species. Blue: present in *M. tuberculosis*, *M. bovis* and *M. bovis* BCG, Red: present in *M. tuberculosis* and *M. bovis* [33], Green: present in *M. tuberculosis* and *M. bovis* BCG [3,18,19], Purple: present in *M. tuberculosis* [44].. *Acon*: aconitase, *Cgl*: cystathionine gamma-lyase *Cit*: citrate synthase, *CysE, CysK1, CysK2*: cysteine synthase*, CysM*: cystathionine beta-synthase, *Eno*: enolase, *Gdh*: glycine dehydrogenase, *GlcB*: malate synthase, *GlyA*: glycine hydromethytransferase, *Gpm*: phosphoglycerate mutase, *Icl*: isocitrate lyase, *Mdh*: malate dehydrogenase, *Pdh*: pyruvate dehydrogenase, *PykA*: pyruvate kinase, *Ppdk*: pyruvate phosphate dikinase, *SerA*: phosphoglycerate dehydrogenase, *SerB*: phosphoserine phosphatase, *SerC*: phosphoserine transaminase, *SdaA*: serine deaminase.

The amino acid data regarding serine metabolism was of particular interest because the results differed between the three mycobacteria and appeared inconsistent with *in silico* simulations. *In silico* serine metabolism is relatively complex compared to other amino acids as multiple pathways converge around serine (Figure 2). However, in *vitro* it is likely only the interconversion of pyruvate and serine (serine dehydratase) enables utilisation of serine as a sole nitrogen source: as inadequate expression of serine dehydratase results in the inability of some BCG strains, including *M. bovis* BCG Pasteur, to utilise serine as a nitrogen source [33]. *M. tuberculosis*, which has been found to adequately express serine dehydratase can utilise L-serine [33,52], as can *M. bovis* strains (Table 6). *In silico*, however, the GSMN-BCG inaccurately predicts that serine is a viable sole nitrogen source for *M. bovis* BCG with flux to glycine (serine hydromethyltransferase) enabling viability. The three mycobacteria used in this study have two serine hydromethyltransferases (*glyA1*and *glyA2*); with enzyme activity demonstrated in *M. tuberculosis* [54]. The *glyA1* of *M. bovis* and *M. bovis* BCG have an amino acid substitution as compared to *M. tuberculosis* although the effect on the enzyme activity has not been tested. If the interconversion of serine and glycine is feasible in *M. bovis* BCG, this reaction doesn’t appear to take place when serine is supplied as sole nitrogen source. However, because serine was shown to be a viable sole carbon source for *M. tuberculosis, M. bovis* and *M. bovis* BCG, flux from serine must be able to enter the TCA cycle. The most probable route for this would be via glycine (Figure 2).

### Validation of the model by comparison with *in vitro* growth rates

The GSMN-BCG was used to predict cellular growth rates on Roisin’s media using published experimental data [29] on substrate uptake rates and corresponding growth rates. Overall, *in silico* predictions were similar to the experimentally-determined growth rates (Table 7) and the growth rate generated by the GSMN-TB 1.1 network. Due to the inability of *M. bovis* to utilise glycerol, GSMN-MB was not tested.

**Table 7:** Comparison of *in silico* and *in vitro* growth rates on Roisin’s minimal media using calculated substrate uptake rates.

		*M. bovis* BCG		*M. tuberculosis*
Substrate	Specific consumption rate (mmol g biomass-1 h-1)	*in vitro* growth rate	*in silico* growth rate	*in silico* growth rate
Glycerol	0.39	0.010	0.009	0.010
Tween 80	0.002			
Glycerol	0.74	0.030	0.030	0.030
Tween 80	0.09			

### Validation of the model by comparison with global mutagenesis data

*In silico* gene essentiality predictions were compared with *in vitro* gene essentiality data as determined by Transposon site hybridization (TraSH) mutagenesis [30] and deep-sequencing [31] (Tables S5-S7). All models gave a very similar predictive accuracy (76-77%; Table 8) when compared against TraSH data, however, results were only available for 82% of genes in the models. Comparison of predictions with a more comprehensive evaluation (100% network coverage) of gene essentiality by deep-sequencing also generated a similar predictive accuracy (75%; Table 8).

**Table 8:** Accuracy of *in silico* gene essentiality predictions.

	TraSH			Deep sequencing		
Category	GSMN-TB 1.1	GSMN-MB	GSMN-BCG	GSMN-TB 1.1	GSMN-MB	GSMN-BCG
True positive	23%	24%	24%	23%	24%	24%
False positive	8%	8%	8%	6%	6%	6%
False negative	16%	16%	16%	19%	19%	20%
True negative	53%	51%	51%	52%	50%	50%
Correct predictions	77%	76%	76%	75%	75%	75%
p value	0.005	0.011	0.008	0.955	0.687	0.863

Percentage of *in silico* gene essentiality predictions categorised as: true-positive: essential both *in silico* and *in vitro*; false-positive: essential in silico, nonessential *in vitro*; true-negative: nonessential *in silico* and *in vitro*; false-negative: nonessential *in silico*, essential *in vitro*

**Figure 3:**
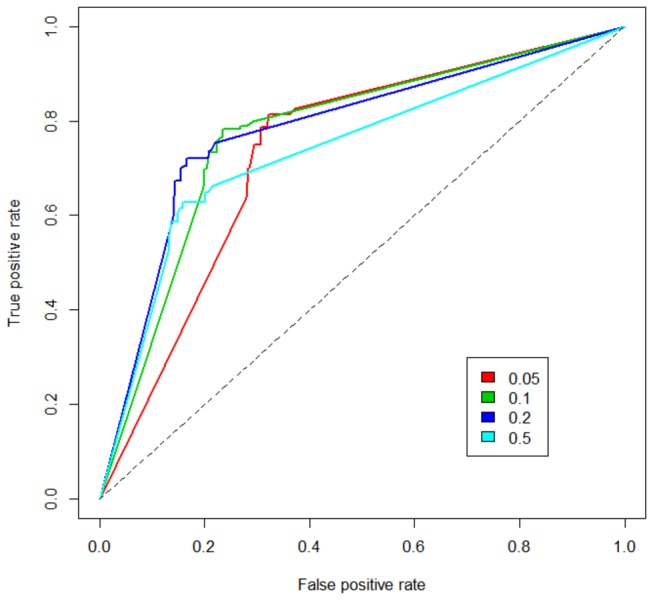
The GSMN-MB ROC curve for TraSH thresholds. The plot shows ROC curves for different transposon site hybridisation (TraSH) ratio thresholds for the determination of essential genes in experimental data [30]. Five ROCs are plotted with 4 different TraSH thresholds as shown in the legend box. Each ROC curve shows the points corresponding to True positive rate (sensitivity) and false positive rate (1-specificity) of the model predictions obtained for all growth rate thresholds. For all ROC curves see Figures S1-S5.

Designation of a gene as either essential or non-essential is a binary characteristic that is generated from a continuous measurement of growth rate or mutant abundance (determined by microarray or sequencing) by applying an arbitrary cut-off value. To examine the influence of the cut-off value on predictive accuracy we plotted Receiver Operating Characteristic (ROC) curves (Figure 3; Figure S1-5). The majority (~91%) of *in silico* mutants generated a predicted growth rate equal to the wild-type or zero, so variation in the *in silico* growth rate threshold had very little influence on the result. However, in *vitro* cut-offs did influence prediction accuracy so ROC curves could be used to identify optimal values for the cut-off value of the *in vitro* measurement signal. For both TraSH and deep sequencing datasets the optimal microarray and p-value cut-offs were the original values of 0.2 and 0.05 respectively.

Examining different areas of metabolism, the predictive accuracy (using deep sequencing data) varied from 61-62% in central carbon metabolism (glycolysis, TCA cycle, pentose phosphate cycle, methylcitrate cycle and anaplerotic reactions) to 95-96% in β-oxidation of fatty acids (Table 9). The chief source of errors for central carbon metabolism genes were false-negatives: genes predicted to be non-essential *in silico* but experimentally found to be essential. This likely reflects the multiple alternative pathways available for flux in *in silico* central metabolism, many of which are likely to be incapable of supporting sufficient flux for growth in real organisms. Peripheral areas of the network tend to generate higher predictive accuracies as fewer alternative pathways are available. Sources of false-negative *in silico* predictions may be due cross feeding between mutants in global mutagenesis studies.

**Table 9:** Percentage accuracy of gene essentiality predictions for each reaction pathway within the reaction networks.

Pathway	GSMN-TB 1.1	GSMN-MB	GSMN-BCG
Amino acid metabolism	76	75	75
Carbohydrate metabolism (excl. central metabolism)	72	75	71
Cell wall synthesis	88	88	88
Central carbon metabolism	61	62	61
Cofactor biosynthesis	66	66	66
Lipid biosynthesis	77	77	78
Nucleotide biosynthesis	70	66	66
Other functions	65	63	63
Transport reactions	94	95	95
β-oxidation of fatty acids	95	96	96

## Discussion

The first genome scale metabolic models for *M. bovis* and *M. bovis* BCG, GSMN-MB and BCG, instantiate current metabolic knowledge and deliver a high degree of accuracy for predicting *in vitro* data. In combination with a *M. tuberculosis* network we have demonstrated how these models, and their application to interrogate high-throughput experimental data, shed new light on metabolic differences between the vaccine, bovine and human strains of the tubercle bacillus. Therefore, these reaction networks successfully simulate many aspects of mycobacterial metabolism, and provide an invaluable tool for the investigation of metabolic differences between these strains.

*In silico* models are essentially a mathematical instantiation of current knowledge. Interrogating the models to generate predictions that can be tested against experimental datasets is thereby an efficient means of probing inconsistencies and limitations of current knowledge. The study described here illustrates this approach. The discordance between *in silico* and *in vitro* phenotypes highlights the limitations of FBA predictions in which any route for flux from substrates to products may be utilised by the solution. For instance, the model predicted growth of *M. bovis* on glucose. This is contrary to expectations; indeed, this inability is a distinguishing feature of the *M. bovis* strain of the tubercle bacillus. Presumably the pathways utilised *in silico* to connect between glycolysis and the TCA cycle are not able to support growth *in vitro* [18,19] and additional defects [18], such as the identified deficiency in glucose uptake are also contributing to this phenotype.

However, our studies show that despite being unable to utilise glucose as a sole carbon source, the sugar can be assimilated by *M. bovis*. Indeed, glucose assimilation was stimulated by addition of Tween 80 to the glucose media [8,53]. Tween 80 is hydrolysed by mycobacteria to release fatty acids, such as oleic acid, that may be oxidised to acetate and thereby enter the TCA cycle to support the synthesis of essential metabolic precursors, such as oxaloacetate and α-ketoglutarate, as well as providing substrates for energy generation. The result suggests that *M. bovis* is capable of assimilating carbohydrates such as glucose, but not delivering carbon from those substrates to the TCA cycle [18]; as the additional glucose was not oxidised to CO_2_ and was presumably incorporated directly into cell biomass. The result is consistent with a previous study that demonstrated compartmentalisation during co-utilisation of different substrates in *M. tuberculosis* [55]. When supplied with both carbohydrate (such as glucose) and fatty acid (acetate), the carbohydrate was assimilated via glycolysis and the pentose phosphate cycle and mostly incorporated into biomass; whereas acetate was mainly used for energy generation via the TCA cycle [55]. It is interesting that this disconnect is unidirectional: *M. bovis* can utilise acetate as a sole carbon source so must be able to drive flux from acetate to glucose via gluconeogenic pathways.

Interrogating *in silico* models with high throughput phenotype and gene essentiality data provides a powerful route towards refinement of the networks, but also provides an insight into the complex relationship between genome and phenotype. The mycobacterial networks are well suited for investigations into the system-wide effects of genetic mutations due to their close evolutionary history, high genetic similarly and diverse phenotypes [3,5,6,8,50]. Both phenotype and gene essentiality data were predicted with a high degree of accuracy, but interestingly key discrepancies between existing genetic knowledge and experimental phenotypes were observed. For instance, the *in silico* models predicted very few metabolic differences between *M. tuberculosis, M. bovis* and *M. bovis* BCG, yet substrate utilisation capability between the strains decreased from *M. tuberculosis* > *M. bovis* and *M. bovis* BCG. It seems that during their evolutionary passage from their common ancestor (that is presumed to be closer to *M. tuberculosis*) [50], *M. bovis* and *M. bovis* BCG have lost some of their capability to metabolise compounds. The basis of this loss in metabolic versatility is unknown but could result from differences in enzyme regulation. For instance, the loss of pyruvate kinase in *M. bovis* results in a global alteration of enzyme expression which reroutes the catabolic pathways of metabolic substrates [19]. 

By comprehensively testing the utilisation of amino acids as sole nitrogen sources, loss in metabolic versatility was particularly evidenced in this study. *M*. *tuberculosis*, *M*. *bovis* and *M*. *bovis* BCG were able to assimilate nitrogen from 12, 8 and 6 amino acids respectively, despite current knowledge resulting in almost identical *in silico* predictions. The only difference *in silico* is the ability of *M*. *tuberculosis* to utilise alanine as a nitrogen (and carbon) source in contrast to *M*. *bovis* and *M*. *bovis* BCG. This difference corresponds to the previously mentioned mutation in the alanine dehydrogenase gene in *M*. *bovis* and *M*. *bovis* BCG, indicating that, in accordance with previous studies [33,44] this gene is required for alanine assimilation. Another interesting finding was that some amino acids, such as serine, could act as carbon but not nitrogen sources, indicating that the pathways responsible for amino acid degradation differ for carbon and nitrogen assimilation.

The differences observed between *in silico* and *in vitro* data not only identify areas of metabolism which require further investigation, but enable iterative network modifications. Since the model development process is continuous, the networks are altered as new information becomes available. These genome scale models already successfully simulate many aspects of mycobacterial growth and metabolism, but it is to be expected that the networks will gradually become more representative of cellular metabolism over time. Further comparative analysis will help to uncover the genetic basis for the observed phenotypic and pathogenic differences between these mycobacteria, and stimulate new approaches to the control of these diseases, such as the development of novel vaccines.

## Supporting Information

Figure S1 **GSMN-TB 1.1 TraSH ROC curve.**
(TIFF)

Figure S2 **GSMN-BCG TraSH ROC curve.**
(TIFF)

Figure S3 **GSMN-TB 1.1 Deep sequencing ROC curve.**
(TIFF)

Figure S4 **GSMN-MB Deep sequencing ROC curve.**
(TIFF)

Figure S5 **GSMN-BCG Deep sequencing ROC curve.**
(TIFF)

Table S1 **GSMN-TB 1.1.**
(XLS)

Table S2 GSMN-MB.
(XLS)

Table S3 GSMN-BCG.
(XLS)

Table S4 **Alterations to GSMN-TB 1.1 to create GSMN-MB and BCG networks.**
(XLS)

Table S5 **GSMN-MB gene essentiality.**
(XLSX)

Table S6 **GSMN-BCG gene essentiality.**
(XLSX)

Table S7 **GSMN-TB 1.1 gene essentiality.**
(XLSX)

Table S8 ***M. tuberculosis* carbon utilisation.**
(XLSX)

Table S9 ***M. bovis* carbon utilisation.**
(XLSX)

Table S10 ***M. bovis* BCG carbon utilisation.**
(XLSX)

Table S11 ***M. tuberculosis* nitrogen utilisation.**
(XLSX)

Table S12 ***M. bovis* nitrogen utilisation.**
(XLSX)

Table S13 ***M. bovis* BCG nitrogen utilisation.**
(XLSX)

Model S1 **GSMN-TB 1.1**
(XML)

Model S2 **GSMN-MB**
(XML)

Model S3 **GSMN-BCG**
(XML)
