# Supplementary figures and images for: Systems-Based Approaches to Probing Metabolic Variation within the Mycobacterium tuberculosis Complex
Source: PLoS One. 2013 Sep 17;8(9):e75913. doi: 10.1371/journal.pone.0075913 (PMC3783153; doi:10.1371/journal.pone.0075913)

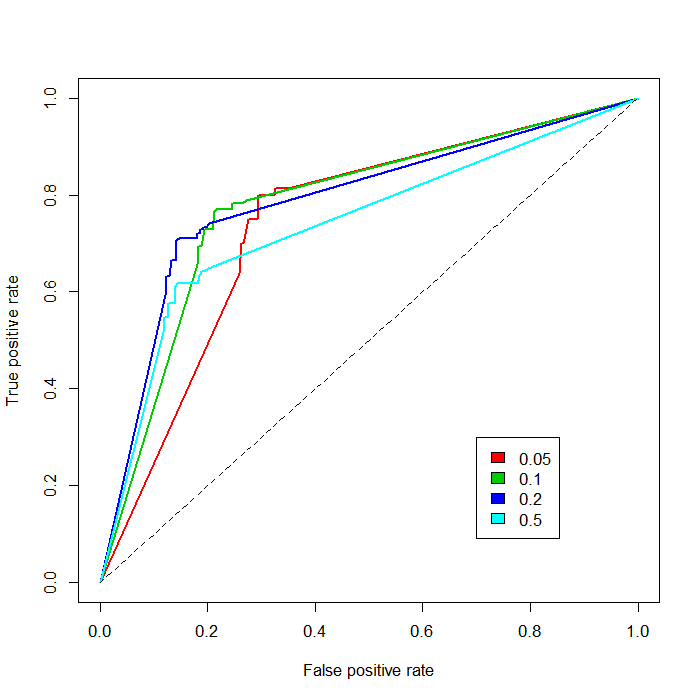

Supplement: Figure S1 — GSMN-TB 1.1 TraSH ROC curve. (TIFF) [file pone.0075913.s001.tiff]

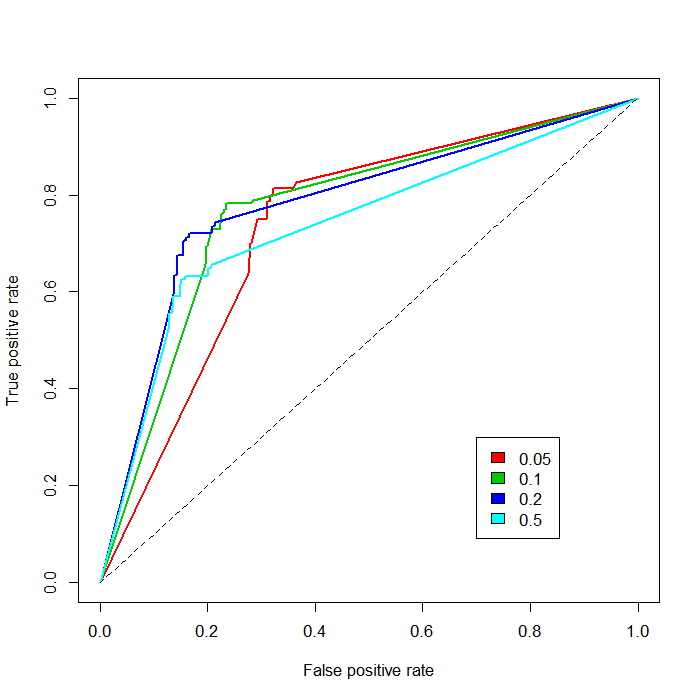

Supplement: Figure S2 — GSMN-BCG TraSH ROC curve. (TIFF) [file pone.0075913.s002.tiff]

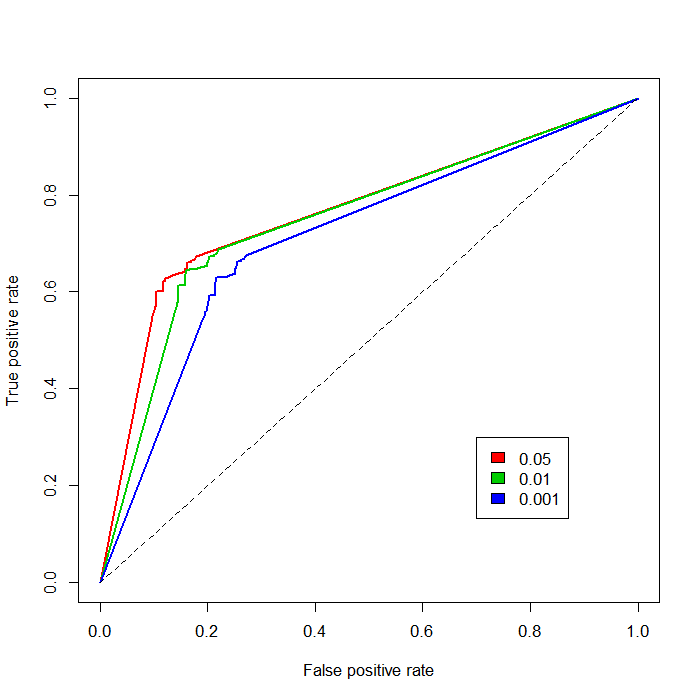

Supplement: Figure S3 — GSMN-TB 1.1 Deep sequencing ROC curve. (TIFF) [file pone.0075913.s003.tiff]

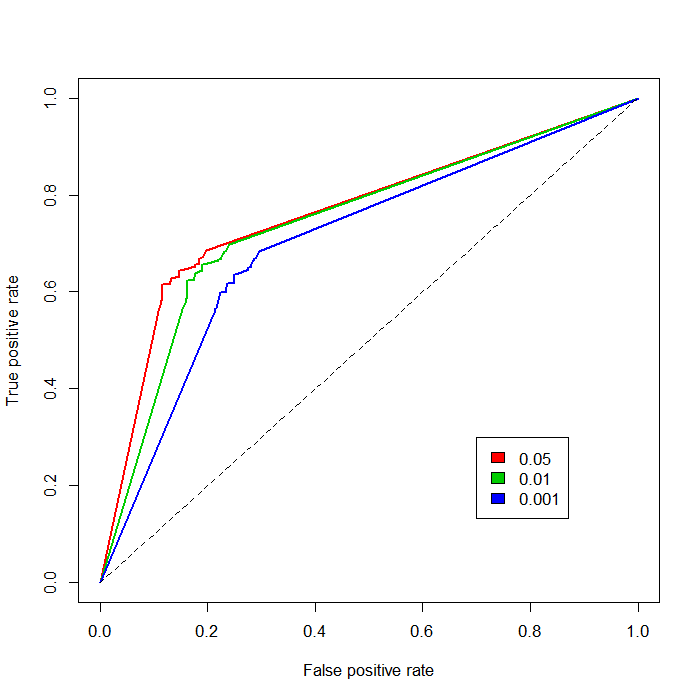

Supplement: Figure S4 — GSMN-MB Deep sequencing ROC curve. (TIFF) [file pone.0075913.s004.tiff]

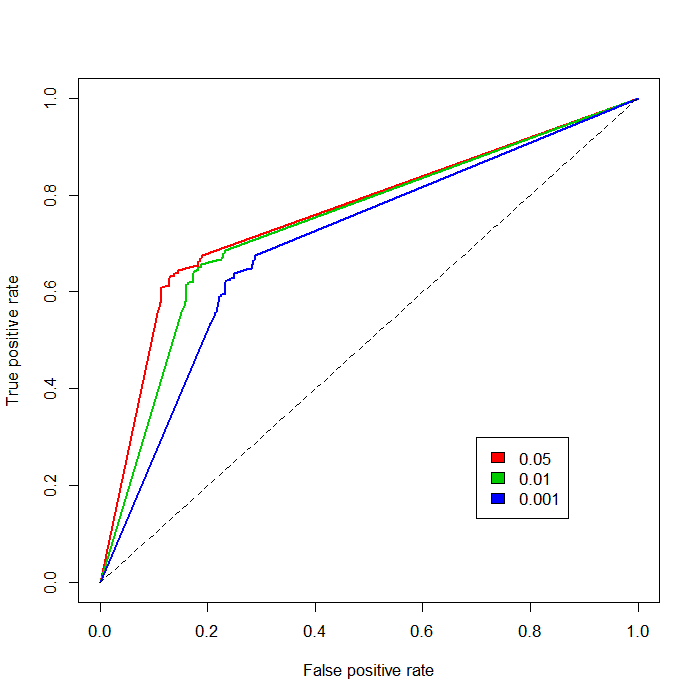

Supplement: Figure S5 — GSMN-BCG Deep sequencing ROC curve. (TIFF) [file pone.0075913.s005.tiff]
